# Intimate Partner Violence and Depression Symptom Severity among South African Women during Pregnancy and Postpartum: Population-Based Prospective Cohort Study

**DOI:** 10.1371/journal.pmed.1001943

**Published:** 2016-01-19

**Authors:** Alexander C. Tsai, Mark Tomlinson, W. Scott Comulada, Mary Jane Rotheram-Borus

**Affiliations:** 1 Massachusetts General Hospital, MGH Global Health, Boston, Massachusetts, United States of America; 2 Harvard Center for Population and Development Studies, Cambridge, Massachusetts, United States of America; 3 Mbarara University of Science and Technology, Mbarara, Uganda; 4 Stellenbosch University, Stellenbosch, South Africa; 5 Center for HIV Identification, Prevention and Treatment Services, University of California at Los Angeles, Los Angeles, California, United States of America; 6 Department of Psychiatry and Biobehavioral Sciences, Semel Institute for Neuroscience and Human Behavior, University of California at Los Angeles, Los Angeles, California, United States of America; Western Sydney University, AUSTRALIA

## Abstract

**Background:**

Violence against women by intimate partners remains unacceptably common worldwide. The evidence base for the assumed psychological impacts of intimate partner violence (IPV) is derived primarily from studies conducted in high-income countries. A recently published systematic review identified 13 studies linking IPV to incident depression, none of which were conducted in sub-Saharan Africa. To address this gap in the literature, we analyzed longitudinal data collected during the course of a 3-y cluster-randomized trial with the aim of estimating the association between IPV and depression symptom severity.

**Methods and Findings:**

We conducted a secondary analysis of population-based, longitudinal data collected from 1,238 pregnant women during a 3-y cluster-randomized trial of a home visiting intervention in Cape Town, South Africa. Surveys were conducted at baseline, 6 mo, 18 mo, and 36 mo (85% retention). The primary explanatory variable of interest was exposure to four types of physical IPV in the past year. Depression symptom severity was measured using the Xhosa version of the ten-item Edinburgh Postnatal Depression Scale. In a pooled cross-sectional multivariable regression model adjusting for potentially confounding time-fixed and time-varying covariates, lagged IPV intensity had a statistically significant association with depression symptom severity (regression coefficient b = 1.04; 95% CI, 0.61–1.47), with estimates from a quantile regression model showing greater adverse impacts at the upper end of the conditional depression distribution. Fitting a fixed effects regression model accounting for all time-invariant confounding (e.g., history of childhood sexual abuse) yielded similar findings (b = 1.54; 95% CI, 1.13–1.96). The magnitudes of the coefficients indicated that a one–standard-deviation increase in IPV intensity was associated with a 12.3% relative increase in depression symptom severity over the same time period. The most important limitations of our study include exposure assessment that lacked measurement of sexual violence, which could have caused us to underestimate the severity of exposure; the extended latency period in the lagged analysis, which could have caused us to underestimate the strength of the association; and outcome assessment that was limited to the use of a screening instrument for depression symptom severity.

**Conclusions:**

In this secondary analysis of data from a population-based, 3-y cluster-randomized controlled trial, IPV had a statistically significant association with depression symptom severity. The estimated associations were relatively large in magnitude, consistent with findings from high-income countries, and robust to potential confounding by time-invariant factors. Intensive health sector responses to reduce IPV and improve women’s mental health should be explored.

## Abstract 2

In a population-based prospective cohort study, Alexander C. Tsai and colleagues examine bidirectional associations between intimate partner violence and depressed mood among pregnant and postpartum women living near Cape Town, South Africa.

## Editors' Summary

**Background:**

Violence against women perpetrated by an intimate partner is a common and widespread problem. Rates of intimate partner violence (IPV, also called domestic violence) vary widely between countries but, globally, nearly a third of women experience IPV at some time in their life. IPV is defined as physical, sexual, or emotional violence that is perpetrated on an individual by a current or former partner or spouse. Physical violence includes slapping, pushing or shoving, hitting with a fist or another object, and threatening or attacking a partner with a weapon; sexual violence means forcing a partner to take part in a sex act when the partner does not give consent; and emotional violence includes threatening a partner by, for example, preventing them seeing their family. The adverse effects of IPV against women include physical injury and sexual and reproductive health problems such as HIV infection and unwanted pregnancies.

**Why Was This Study Done?:**

Studies undertaken in high-income countries have also shown an association between IPV and adverse mental health outcomes among women, such as depression (long-lasting and overwhelming feelings of sadness and hopelessness) and suicidal behavior. However, few if any studies on the association between IPV and depression have been conducted in sub-Saharan Africa, where the rates of IPV against women are among the highest in the world. In this population-based prospective cohort study, the researchers analyze longitudinal data collected during a cluster-randomized trial that involved more than 1,200 women living in townships near Cape Town, South Africa. The primary aim of this randomized trial was to investigate whether regular visits by “mentor mothers” (women who had successfully raised children in the face of adversity) improved maternal and child health in the 3 y following the child’s birth. As part of this trial, the researchers collected data at multiple time points (longitudinal data) on women’s experiences of IPV and symptoms of depression. Here, the researchers conduct a secondary analysis of these data to estimate the association between IPV severity and depression symptom severity among women during and following pregnancy.

**What Did the Researchers Do and Find?:**

During the cluster-randomized trial, the researchers asked the women about their exposure to physical IPV during the past year in surveys undertaken at baseline, 6 mo, 18 mo, and 36 mo. Depression symptom severity was also measured in the women at these time points using a version of the ten-item Edinburgh Postnatal Depression Scale in the local language Xhosa; this scale is an instrument that screens for postnatal depression by asking questions about depressive symptoms experienced during the past seven days by women who have recently had a baby, such as how often they have felt sad or miserable. Statistical analyses of these data indicated that, after allowing for other factors that might affect depression symptom severity, IPV intensity had a statistically significant association (an association unlikely to have arisen by chance) with depression symptom severity. That is, an increase in IPV severity among the study participants was associated with an increase in depression symptom severity over the same period. Notably, this association was bidirectional. That is, depression symptom severity also had a statistically significant association with IPV intensity.

**What Do These Findings Mean?:**

These findings show that, among women living in poor neighborhoods in Cape Town who have recently had a baby, IPV severity had a statistically significant association with depression symptom severity. The magnitude of this association was relatively large and consistent with findings from high-income countries. The accuracy of these findings may be limited by certain aspects of this study. For example, because the study participants were not asked about sexual violence, the severity of IPV exposure may be underestimated. Nevertheless, these findings—in particular, the demonstration that the association between IPV and depression is bilateral—have important policy and programmatic implications for women’s health in sub-Saharan Africa. Specifically, because IPV and depression may be intertwined in a vicious cycle, with IPV increasing the risk of future depression and depression increasing the risk of future victimization, multi-component interventions that combine a broad-based package of services (for example, provision of legal aid, transitional housing, and childcare support) with interventions designed to treat depression (for example cognitive-behavioral therapy) may be needed to reduce IPV and improve women’s mental health.

**Additional Information:**

This list of resources contains links that can be accessed when viewing the PDF on a device or via the online version of the article at http://dx.doi.org/10.1371/journal.pmed.1001943.

- A *PLOS Medicine* Research Article by Devries et al. reviews the evidence from high-income countries for an association between IPV and depression
- The World Health Organization provides detailed information on intimate partner violence and on depression (some information in several languages)
- The UK National Health Service Choices website provides information about intimate partner violence, including some personal stories, and detailed information about depression
- The US Centers for Disease Control and Prevention provides information about intimate partner violence and links to further resources
- The US National Domestic Violence Hotline provides confidential help and support to people experiencing IPV; its website includes personal stories of IPV
- The US National Institute of Mental Health provides information on all aspects of depression
- MedlinePlus provides links to other resources about domestic violence and about depression (in English and Spanish)
- More information about the "mentor mother" trial is available

## Introduction

Violence against women by intimate partners remains unacceptably common worldwide [1–5], particularly in South Africa, where violence against women occurs at rates that are among the highest in the world [6–9]. The cross-sectional association between intimate partner violence (IPV) and adverse mental health-related outcomes among women is well known [10–12]. These studies are consistent with a broader body of literature linking stressful life events with incident major depressive episodes [13–15], as well as literature linking traumatic stressors to post-traumatic stress disorder and suicide [16–18]. Moreover, acts of violence against women perpetuate gender-unequal norms about the acceptability of violence, which itself compromises women’s reproductive health and decision-making irrespective of any direct exposure to violence [2,19–22].

In a recently published systematic review, Devries and colleagues [23] identified 13 longitudinal studies linking IPV to incident depression, none of which were conducted in sub-Saharan Africa. This is an important gap in the literature because rates of partner and non-partner violence in sub-Saharan Africa are among the highest in the world [4,5]. Methodologically, the importance of conducting longitudinal studies in this field was highlighted by Foa and colleagues [24], whose conceptual model suggests that psychological difficulties may be a risk factor for subsequent victimization. For example, women with poor mental health may selectively partner with men who also have poor mental health (which is a risk factor for perpetration [25]) or have greater difficulty extracting themselves from abusive relationships [26]. Also consistent with this line of inquiry are intervention studies showing that effective depression treatment can reduce the probability of re-victimization [27]. Short of experimental study designs like these, longitudinal studies offer a design superior to cross-sectional studies in their ability to adjust for unobserved confounding and ensure temporal ordering of the exposures and outcomes of interest. Since the publication of the review by Devries and colleagues [23], one recent study showed that sexual violence was associated with depression among HIV-positive women in rural Uganda [28], but interpretation of those findings was limited by the study’s small sample size, relatively unique population, and the observation of relatively few exposure events.

To address these gaps in the literature, we analyzed longitudinal data collected during the course of a 3-y cluster-randomized trial conducted in 2009–2014 with more than 1,200 pregnant women living in 24 neighborhoods near Cape Town, South Africa. The primary aim of the randomized trial was to determine whether a community-based home visiting intervention could improve maternal and child health over 3 y post-birth. With repeated measures of both IPV and depression symptom severity, these data offered us the opportunity to estimate the association between IPV and depression while adjusting for both observed and unobserved confounding.

## Materials and Methods

### Ethics Statement

All interviews were conducted in accordance with ethical and safety recommendations promulgated by the World Health Organization [29]. Namely, research assistants were trained on how to administer surveys for gathering sensitive information and provided assurances of confidentiality. The survey was framed generally as being part of a study of family health and well-being, not a study about violence against women. In consultation with on-site supervisors, research assistants provided referrals to local counseling resources and/or child social services as needed, with standardized protocols in place to refer women to emergency services in the case of acutely elevated risk of harm to self or harm from others. All study procedures were approved by the South General Institutional Review Board of the University of California at Los Angeles and the Health Research Ethics Committee of the Stellenbosch University Faculty of Health Sciences. A four-person Data Safety Monitoring Board populated by local and international experts monitored implementation of the study. The secondary analysis described in this manuscript was based on a de-identified dataset and did not require additional approval or consent.

### Study Population

Details of the study design, field training, and primary outcome analyses at 6, 18, and 36 mo have already been published [30–35] (ClinicalTrials.gov registration NCT00972699). The study was conducted during 2009–2014 in three townships surrounding Cape Town, South Africa. All pregnant women living in 24 neighborhoods (matched on population density, number of bars, distance to health care, and access to public works infrastructure) were identified and recruited into the study, with a 98% participation rate. These matched neighborhoods were randomized in blocks of four to either a home visiting intervention or standard clinic care groups. Standard clinic care was available (within 5 kilometers) to all women living in the study catchment area and generally consisted of tuberculosis and HIV testing, partner HIV testing, antiretroviral therapy, antenatal and postnatal care, well-child clinics, and primary health care [30].

The home visiting intervention was implemented by the Philani Maternal, Child Health, and Nutrition Project, a non-governmental organization that has been operating in the Western Cape of South Africa since 1979 [36] and which has since expanded to the Eastern Cape of South Africa as well as Ethiopia and Swaziland. Philani implements a “mentor mother” program, recruiting women from the community who have successfully raised thriving children despite concentrated adversity and then training these women to serve as paraprofessional community health workers for home visiting among pregnant women and their families [37,38]. For the purposes of the randomized trial, the Philani intervention was standardized and augmented with training in a pragmatic model of problem-solving and cognitive-behavioral techniques to address major community health challenges, including HIV/tuberculosis, malnutrition, and alcohol use [39,40,41]. An independent team of Xhosa-speaking research assistants obtained written informed consent from all study participants and collected survey data through face-to-face interviews conducted at baseline, 6 mo, 18 mo, and 36 mo. Analyses of these data revealed that the “Philani Plus” intervention improved overall maternal and child health across a number of different outcomes, notably those related to HIV-prevention behaviors, breastfeeding, and child growth over 18 mo; and maternal emotional well-being, child language development, and child growth over 36 mo [31–35].

### Measures

The primary outcome of interest in this secondary analysis was depression symptom severity, which was measured at all time points with the Xhosa version of the ten-item Edinburgh Postnatal Depression Scale (EPDS) [42]. Scale items inquire about depressive symptoms within a 7-d recall period, with responses scored on a four-point Likert-type scale ranging from 0 (“not at all”) to 3 (“all the time”). Among Xhosa-speaking women, the EPDS has been shown to have a coherent internal structure [43], high sensitivity and specificity for detecting major depressive disorder [44–46], and good construct validity [39,47]. In the baseline sample, the EPDS had good internal consistency (Cronbach’s alpha = 0.89), and, using 500 bootstrap replications to compute the standard error, the 95% confidence interval (CI) was 0.88–0.90.

The primary explanatory variable of interest in this secondary analysis was experience of IPV, measured with a four-item scale. Following Straus’ [48] approach of asking behaviorally specific questions, the IPV scale included items inquiring about the frequency with which a woman’s current or previous intimate partner had, during the past 12 mo, slapped or thrown anything at her; pushed or shoved her; hit her with a fist or another object; or threatened or attacked her with a gun, knife, or other weapon. Responses were scored on a four-point Likert-type scale ranging from 1 (“never”) to 4 (“many”). Together, these four items had acceptable internal consistency, with a Cronbach’s alpha of 0.75 (95% CI, 0.71–0.80) in the baseline sample. To generate an omnibus measure of the intensity of IPV across all four items, following Kling and colleagues [49] we defined a summary IPV index as the equally weighted average of the four *z*-scores (i.e., each item was standardized to a mean of 0 and standard deviation of 1, and then the summary index was defined as their average value). While the absolute values of the index carry no meaning, higher values denote greater intensity of IPV.

We adjusted our estimates of the association between IPV intensity and depression symptom severity for a number of potentially confounding time-invariant and time-varying covariates. Time-invariant covariates were elicited at the baseline interview and included binary indicators denoting whether the participant had been assigned to the intervention or standard clinic care arm, age at baseline, and whether the participant had completed high school. Household asset wealth was elicited by asking participants a series of 13 questions about household assets and housing characteristics (e.g., whether there is a flush toilet in the home, whether a household member owns a radio, etc.). Then, following the method of Filmer and Pritchett [50], we applied principal components analysis to these variables. The first principal component was retained and used to define the asset wealth index, and participants were sorted into quintiles of relative asset wealth.

Time-varying covariates were elicited at each interview. Time elapsed since the baseline interview was measured in months. We included binary indicators denoting whether the participant was employed (either full- or part-time), whether the father of the child was staying with the participant, HIV serostatus (classified as HIV-positive, HIV-negative, or unknown/refused testing), and whether the participant had been diagnosed with high blood pressure or diabetes. Household monthly income was measured in South African Rand. Alcohol abuse was measured with the three-item consumption subset of the Alcohol Use Disorders Identification Test (AUDIT-C) [51–53].

### Statistical Analysis

We did not publish or pre-register a plan for this secondary analysis. The analysis plan is described below, with any deviations noted in S1 Text. Given the repeated-measures design, we sought to estimate the association between IPV intensity and depression symptom severity, adjusted for the time-invariant and time-varying covariates described above. We fitted a linear regression model to the pooled cross-sectional data, specifying the EPDS score as the continuous dependent variable, using cluster-correlated robust estimates of variance [54–56] to correct standard errors for clustering within participants over time. To ensure the correct temporal sequence of the exposure and outcome, IPV measurements were lagged by one time point (an average of 12 mo). The estimated regression coefficients therefore provided information about the association between IPV intensity at one time point and depression symptom severity at the subsequent time point. We sought to determine whether the adverse impacts of IPV were experienced to a greater extent by women at the upper end of the conditional depression distribution. To do this, we fitted quantile regression models [57] to estimate the association between IPV intensity and the 20th, 40th, 60th, and 80th percentiles of the conditional depression distribution, using a covariance matrix of the asymptotic distribution of the quantile regression estimator that permits within-participant correlation over time [58].

Although the regression models included adjustment for a number of potentially important confounding variables, it is possible that some important variables were not observed. For example, in the specific setting of this randomized trial, no data on participants’ histories of child sexual abuse were obtained. Child sexual abuse could potentially confound our estimates of the association between IPV intensity and depression symptom severity [23]; even in the lagged-covariate models where the exposure precedes the outcome, the confounding influence of child sexual abuse would precede both the exposure and the outcome. Estimates could be similarly biased by omitting other types of childhood adversities, such as paternal incarceration or orphanhood [59,60]. We therefore fitted a fixed effects regression model to the data, using within-participant variation over time to identify the estimated associations [61]. The estimated regression coefficients are interpreted as providing information about the association between changes in IPV intensity and changes in depression symptom severity. A substantial advantage of the fixed effects regression model is that the procedure adjusts for confounding, whether observed or unobserved, that is time-invariant over the period of study (such as history of child abuse). The principal disadvantage of the fixed effects regression model is that, because the fixed effects sweep out all time-invariant confounding, only associations between changes in the outcome and changes in time-varying covariates can be examined. Because time-invariant covariates, by definition, do not change over time, they are eliminated from the model. To determine whether changes in IPV intensity were differentially associated with changes in depression symptom severity at different points in the conditional depression distribution, we used Canay’s [62] fixed effects quantile regression model.

### Sensitivity Analyses

We conducted a number of ancillary analyses to assess the robustness of our main findings. First, to confirm that our findings were robust to the specification of the IPV variable, we generated dichotomous exposure variables indicating the presence or absence of any exposure to each of the four types of IPV. These four dichotomous exposure variables were included in the multivariable regression models both independently and jointly as lagged covariates. Second, because caseness for (probable) depression is frequently of clinical interest, we defined probable depression as EPDS ≥13 [45,46,63–65]. We fitted logistic regression models as above, instead specifying probable depression as the binary dependent variable. We also report marginal effects [66] so that the logistic regression coefficients can be interpreted as the percentage-point probability difference in the outcome associated with the covariates. Third, to determine the extent to which the association was potentially bidirectional [23,67], we re-fitted the regression model with IPV intensity as the dependent variable and depression symptom severity as a lagged covariate, thereby estimating the association between depression symptom severity at one time point and IPV intensity at the subsequent time point.

## Results

### Characteristics of the Sample

Summary statistics for the sample are displayed in Table 1. Of 1,238 women initially randomized, there were 117 mother-child dyads in which either the mother or the child died, and these were removed from the study (and, therefore, this analysis) [68]. Of these, 958 (85%) remained at 36-mo follow-up. Women lost to follow-up had a lower median EPDS (7 versus 10; *p* = 0.87 on the non-parametric equality-of-medians test) and were similar on the four types of IPV exposures (*p*-values on χ2 tests ranged from 0.16–0.99). Women lost to follow-up had lower household asset wealth (*p* = 0.06) and were less likely to be employed at baseline (*p* = 0.051) but were otherwise similar on other covariates (*p*-values ranged from 0.16–0.97).

**Table 1:** Baseline characteristics of the sample.

	Standard clinic care arm		Intervention arm		Total	
	N	Pct.	N	Pct.	N	Pct.
Probable depression (EPDS ≥13)	214	36	275	42.7	489	39.5
Partner slapped you, past year						
Never	403	67.8	461	71.6	864	69.8
Once	91	15.3	92	14.3	183	14.8
Few	76	12.8	73	11.3	149	12
Many	24	4	18	2.8	42	3.4
Partner shoved you, past year						
Never	477	80.3	509	79	986	79.6
Once	61	10.3	76	11.8	137	11.1
Few	40	6.7	49	7.6	89	7.2
Many	16	2.7	10	1.6	26	2.1
Partner punched you, past year						
Never	521	87.7	584	90.7	1,105	89.3
Once	37	6.2	28	4.3	65	5.3
Few	22	3.7	28	4.3	50	4
Many	14	2.4	4	0.6	18	1.5
Partner attacked you with weapon, past year						
Never	561	94.6	621	96.4	1,182	95.6
Once	17	2.9	15	2.3	32	2.6
Few	9	1.5	6	0.9	15	1.2
Many	6	1	2	0.3	8	0.6
Age						
18–25 y	300	50.5	323	50.2	623	50.3
26–35 y	257	43.3	270	41.9	527	42.6
≥36 y	37	6.2	51	7.9	88	7.1
Quintile of household asset wealth						
Poorest	116	19.5	140	21.7	256	20.7
Poorer	149	25.1	176	27.3	325	26.3
Middle	105	17.7	109	16.9	214	17.3
Richer	95	16	101	15.7	196	15.8
Richest	129	21.7	118	18.3	247	20
Completed high school	151	25.4	175	27.2	326	26.3
Employed	60	10.1	85	13.2	145	11.7
Father of child stays with participant	311	52.4	362	56.4	673	54.5
HIV serostatus						
HIV-negative	401	67.5	435	67.5	836	67.5
Unknown	47	7.9	60	9.3	107	8.6
HIV Positive	146	24.6	149	23.1	295	23.8
AUDIT-C score, mean (SD)	0.7	2.04	0.65	2.02	0.67	2.03
Monthly household income						
0–499 ZAR	49	8.2	69	10.8	118	9.6
500–1,000 ZAR	88	14.8	88	13.8	176	14.3
1,001–2,000 ZAR	164	27.6	177	27.7	341	27.7
2,001–5,000 ZAR	226	38	226	35.4	452	36.7
5,001–8,000 ZAR	44	7.4	44	6.9	88	7.1
≥8,000 ZAR	9	1.5	10	1.6	19	1.5
Refused to answer	14	2.4	25	3.9	39	3.2
Self-reported diabetes	14	2.4	14	2.2	28	2.3
Self-reported hypertension	61	10.3	62	9.6	123	9.9

AUDIT-C, three-item consumption subset of the Alcohol Use Disorders Identification Test; EPDS, Edinburgh Postnatal Depression Scale; SD, standard deviation; ZAR, South African Rand

For most variables there was a sufficient degree of variation within participants over time. The EPDS had an intra-class correlation (ICC) of 0.19, and the IPV index had an ICC of 0.32, suggesting that most of the total variance was “within group” (over time) rather than “between group.” Intra-class correlation values ranged from 0.12–0.66 for the time-varying covariates, with HIV serostatus (plausibly) featuring the highest intra-class correlation at 0.84.

### IPV Intensity and Depression Symptom Severity

**Fig 1:**
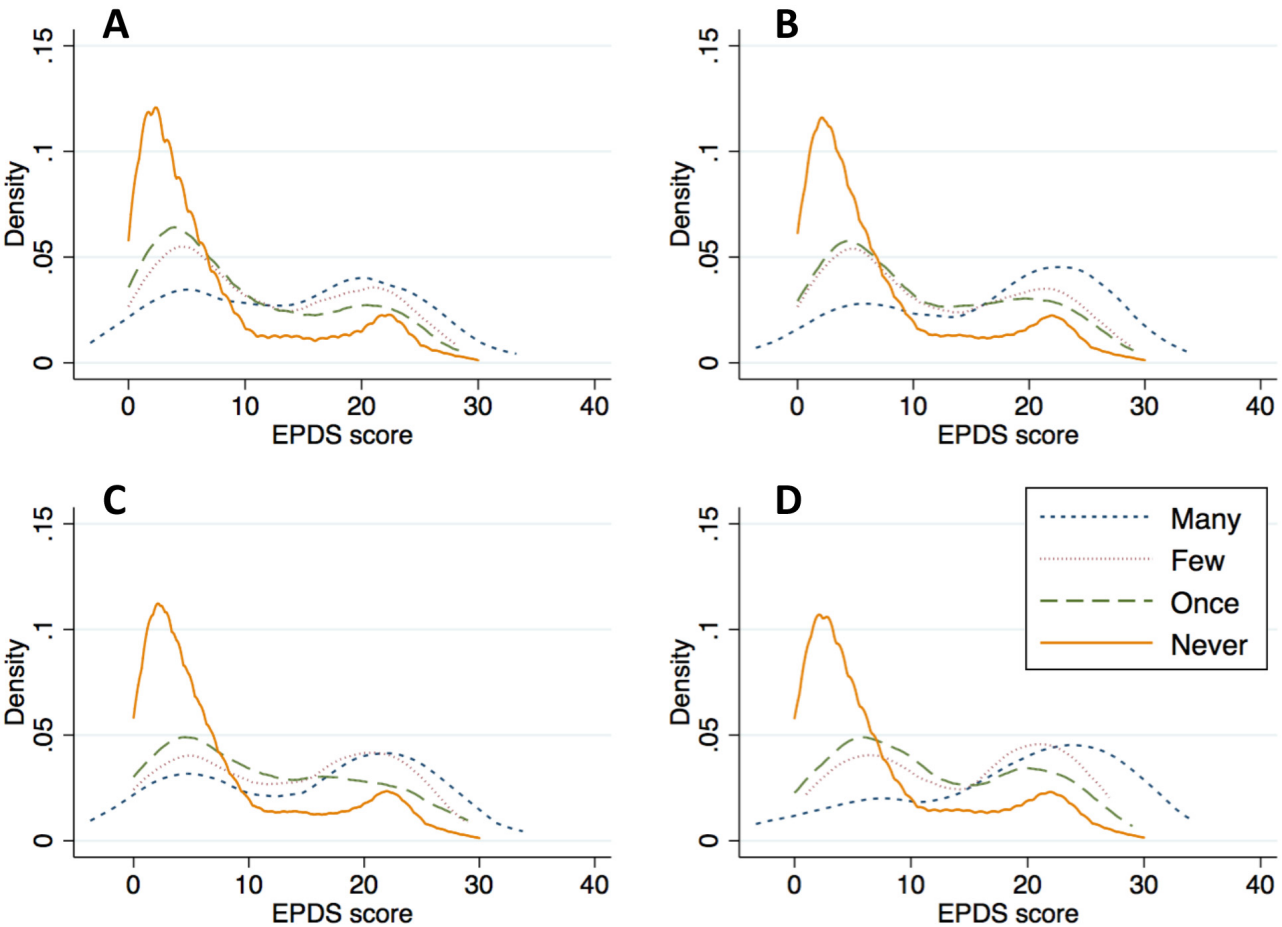
Kernel density plots of depression symptom severity, by type and frequency of intimate partner violence. The scale includes items inquiring about the frequency with which a women’s current or previous intimate partner had, during the past 12 mo, (A) slapped or thrown anything at her; (B) pushed or shoved her; (C) hit her with a fist or another object; or (D) threatened or attacked her with a gun, knife, or other weapon.

At baseline, the prevalence of any IPV varied from 4.4–30.2%, and 39.5% of women screened positive for depression. Kernel density plots suggested that greater frequency of IPV, regardless of type, was associated with greater depression symptom severity (Fig 1). As is apparent in the figure, both the location and shape of the plots suggest the need to investigate changes in the mean and the distribution of the outcome. After multivariable adjustment, IPV intensity had a strong and statistically significant association with depression symptom severity, regardless of the specification. In the pooled cross-sectional analysis, the estimated association between lagged IPV intensity and depression symptom severity was statistically significant (regression coefficient b = 1.04; 95% CI, 0.61–1.47) (Table 2). The quantile regression results, also shown in Table 2, indicate that IPV had an approximately 4- to 5-fold greater impact on depression symptom severity in the upper quintiles of the conditional depression distribution.

**Table 2:** Association between depression symptom severity and lagged intensity of intimate partner violence.

	Mean		Q20		Q40		Q60		Q80	
	coeff.	95% CI	coeff.	95% CI	coeff.	95% CI	coeff.	95% CI	coeff.	95% CI
Index of IPV intensity	1.04	0.61,1.47	0.42	0.25,0.60	0.72	0.44,1.00	1.21	0.74,1.68	2.07	0.99,3.15
Assigned to intervention arm	-0.12	-0.74,0.51	-0.05	-0.26,0.16	-0.18	-0.47,0.11	-0.31	-0.85,0.23	0	-1.95,1.95
Age (per 5 y)	0.36	0.04,0.69	0.1	-0.00,0.21	0.21	0.04,0.38	0.43	0.14,0.72	0.9	-0.22,2.03
Household asset wealth										
Poorest	Ref		Ref		Ref		Ref		Ref	
Poorer	-0.72	-1.72,0.27	-0.35	-0.73,0.03	-0.43	-0.92,0.05	-0.43	-1.29,0.43	-3	-8.75,2.76
Middle	-1.06	-2.16,0.04	-0.57	-0.95,-0.20	-0.79	-1.31,-0.27	-0.72	-1.67,0.24	-3.46	-9.98,3.06
Richer	-0.96	-2.03,0.10	-0.46	-0.88,-0.04	-0.55	-1.07,-0.04	-0.73	-1.63,0.18	-2.02	-8.14,4.11
Richest	-1.23	-2.29,-0.17	-0.47	-0.86,-0.08	-0.75	-1.24,-0.26	-0.89	-1.76,-0.03	-2.95	-9.19,3.29
Completed high school	-1.57	-2.60,-0.54	-0.4	-0.78,-0.02	-0.94	-1.46,-0.42	-0.98	-1.94,-0.01	-3.37	-6.18,-0.56
Time point										
Baseline	Ref		Ref		Ref		Ref		Ref	
6 mo	-0.65	-1.37,0.08	0.05	-0.23,0.33	-0.12	-0.48,0.24	-0.54	-1.24,0.17	-2.56	-5.58,0.46
18 mo	-0.6	-1.34,0.14	-0.49	-0.76,-0.21	-0.93	-1.32,-0.55	-0.76	-1.56,0.04	-0.26	-3.36,2.84
Employed	-1.09	-1.81,-0.38	-0.25	-0.51,0.02	-0.41	-0.72,-0.09	-0.85	-1.36,-0.35	-2.05	-3.95,-0.14
Father of child present	-0.59	-1.28,0.10	-0.25	-0.49,-0.02	-0.44	-0.73,-0.14	-0.84	-1.40,-0.28	-0.61	-3.01,1.78
HIV serostatus										
HIV-negative	Ref		Ref		Ref		Ref		Ref	
Unknown	0.82	-0.94,2.58	0.08	-0.65,0.81	0.18	-0.51,0.87	-0.11	-2.10,1.88	4.2	-4.00,12.40
HIV-positive	0.63	-0.11,1.38	0.23	-0.04,0.50	0.23	-0.12,0.59	0.39	-0.27,1.06	2.45	-1.88,6.77
AUDIT-C score (per point)	0.06	-0.11,0.24	0.04	-0.02,0.10	0.03	-0.07,0.13	0.13	-0.06,0.33	0.26	-0.16,0.68
Monthly household income										
0–499 ZAR	Ref		Ref		Ref		Ref		Ref	
500–1,000 ZAR	0.11	-1.56,1.77	0.19	-0.61,0.99	0	-0.87,0.87	-0.84	-2.95,1.27	2.73	-1.59,7.06
1,001–2,000 ZAR	-0.91	-2.47,0.65	-0.11	-0.86,0.65	-0.48	-1.26,0.30	-1.59	-3.59,0.42	-2.49	-7.69,2.70
2,001–5,000 ZAR	-1.4	-2.96,0.15	-0.36	-1.10,0.37	-0.93	-1.69,-0.16	-2.07	-4.10,-0.04	-3.64	-8.98,1.69
5,001–8,000 ZAR	-2.27	-3.99,-0.54	-0.28	-1.07,0.52	-1.04	-1.85,-0.22	-2.67	-4.71,-0.62	-6.3	-11.94,-0.67
≥8,000 ZAR	-2.74	-4.71,-0.77	-0.61	-1.45,0.22	-1.19	-2.14,-0.25	-2.98	-5.12,-0.84	-6.54	-12.13,-0.95
Self-reported diabetes	2.56	-0.47,5.59	0	-0.92,0.93	1.27	-0.42,2.95	2.17	-3.28,7.62	5.89	-5.53,17.30
Self-reported hypertension	1.05	-0.17,2.26	0.39	-0.01,0.79	0.66	0.09,1.22	0.38	-0.47,1.23	2.79	-0.33,5.90

Q20, Q40, Q60, and Q80 denote quantile regression estimates at the respective percentiles of the conditional distribution of EPDS

AUDIT-C, three-item consumption subset of the Alcohol Use Disorders Identification Test; EPDS, Edinburgh Postnatal Depression Scale; IPV, intimate partner violence; ZAR, South African Rand

In terms of the magnitude of the association, when evaluated at the mean of the covariates, a one-standard deviation increase in IPV intensity was associated with a subsequent 13% relative increase in depressed mood. In multivariable logistic regression, lagged IPV intensity had a statistically significant association with probable depression (adjusted odds ratio [AOR], 1.26; 95% CI, 1.13–1.40). Evaluated at the mean of the covariates, a one–standard-deviation increase in IPV intensity was associated with a 4.0 percentage-point increase in probable depression (a 4.0/39.5 = 10.1% difference relative to the baseline prevalence). When the omnibus IPV exposure variable was replaced with the four IPV exposures as separate covariates, three of the four variables (having been slapped, shoved, or punched) had statistically significant associations with depression symptom severity (all *p* < 0.001, with regression coefficients ranging from 1.85–2.66) (S1 Table). When all four IPV exposures were entered jointly into a multivariable regression model, only being shoved was statistically significant (b = 1.66; 95% CI, 0.61–2.72).

In the fixed effects specification adjusting for all time-invariant confounding, the estimated association between IPV and depression symptom severity remained statistically significant (b = 1.54; 95% CI, 1.13–1.96) (Table 3). In terms of the interpretation of the estimate, a one-standard deviation increase in the intensity of IPV from one time point to the next was associated with a 1.33 point increase in the EPDS over the same time period. Compared to the baseline mean, this value represents a 1.33/10.8 = 12.3% relative increase; compared to the baseline standard deviation, this value represents 1.33/7 = 0.19 standard deviation units. The fixed effects quantile regression estimates, also shown in Table 3, indicate that increases in IPV intensity were associated with the greatest increases in depression symptom severity at the upper end of the conditional depression distribution.

**Table 3:** Association between changes in depression symptom severity and changes in intensity of intimate partner violence.

	Mean		Q20		Q40		Q60		Q80	
	coeff.	95% CI	coeff.	95% CI	coeff.	95% CI	coeff.	95% CI	coeff.	95% CI
Index of IPV intensity	1.54	1.13,1.96	0.96	0.40,1.52	1.37	1.07,1.67	1.98	1.63,2.33	2.32	1.90,2.74
Time point										
Baseline	Ref		Ref		Ref		Ref		Ref	
6 mo	-3.25	-3.85,-2.65	-3.23	-3.95,-2.51	-3.52	-4.01,-3.04	-3.8	-4.32,-3.28	-4.58	-5.60,-3.55
18 mo	-3.76	-4.40,-3.13	-3.8	-4.45,-3.16	-4.08	-4.62,-3.55	-3.92	-4.45,-3.38	-5.11	-6.01,-4.21
36 mo	-4.07	-4.74,-3.39	-4.34	-5.01,-3.68	-4.38	-4.88,-3.88	-4.53	-5.08,-3.98	-5.27	-6.17,-4.37
Employed	0.17	-0.60,0.94	0.39	-0.14,0.92	0.46	0.11,0.82	0.11	-0.19,0.41	-0.14	-0.78,0.51
Father of child present	-0.69	-1.56,0.18	-0.39	-0.90,0.13	-0.35	-0.64,-0.06	-0.86	-1.10,-0.62	-1.02	-1.57,-0.47
HIV serostatus										
HIV-negative	Ref		Ref		Ref		Ref		Ref	
Unknown	-0.25	-1.92,1.41	-0.45	-4.31,3.40	-0.02	-1.16,1.13	-0.2	-0.99,0.60	-2.32	-3.49,-1.16
HIV-positive	-0.08	-1.57,1.41	-0.67	-1.21,-0.14	-0.33	-0.65,-0.01	0.13	-0.14,0.39	1.06	0.27,1.86
AUDIT-C score (per point)	0.18	0.02,0.34	0.12	0.02,0.23	0.14	0.06,0.23	0.18	0.07,0.29	0.31	0.15,0.47
Monthly household income										
0–499 ZAR	Ref		Ref		Ref		Ref		Ref	
500–1,000 ZAR	-1.28	-2.67,0.11	-1.51	-2.77,-0.24	-1.54	-2.45,-0.62	-1.26	-2.40,-0.12	-0.77	-2.58,1.05
1,001–2,000 ZAR	-2.02	-3.34,-0.70	-2.09	-3.25,-0.93	-2.11	-2.97,-1.25	-2.01	-3.00,-1.03	-1.7	-3.44,0.03
2,001–5,000 ZAR	-2.4	-3.71,-1.10	-2.12	-3.22,-1.03	-2.35	-3.15,-1.55	-2.48	-3.41,-1.54	-2.76	-4.38,-1.13
5,001–8,000 ZAR	-2.72	-4.25,-1.18	-1.94	-3.21,-0.67	-2.66	-3.51,-1.80	-2.71	-3.70,-1.72	-3	-4.74,-1.26
≥8,000 ZAR	-2.88	-4.68,-1.07	-2.04	-3.27,-0.82	-2.5	-3.57,-1.43	-2.62	-3.67,-1.56	-3.41	-5.50,-1.32
Self-reported diabetes	-1.16	-3.73,1.42	-1.99	-3.70,-0.27	-0.6	-2.05,0.84	-0.53	-1.81,0.76	-0.92	-2.50,0.66
Self-reported hypertension	0.6	-0.50,1.70	0.15	-0.67,0.96	0.46	-0.20,1.11	0.56	0.10,1.02	1.64	0.12,3.15

Q20, Q40, Q60, and Q80 denote fixed effects quantile regression estimates at the respective percentiles of the conditional distribution of EPDS

AUDIT-C, three-item consumption subset of the Alcohol Use Disorders Identification Test; EPDS, Edinburgh Postnatal Depression Scale; IPV, intimate partner violence; ZAR, South African Rand

When the ordering of the exposure and outcome were reversed, lagged depression symptom severity had a statistically significant association with IPV intensity. Each five-point difference in the EPDS score was associated with a 0.9–2.3 percentage point difference in subsequent IPV risk, depending on the specific outcome, with smaller marginal effects observed for more severe forms of IPV (Table 4). The pattern of smaller marginal effects observed for more severe forms of IPV is due to the lower baseline prevalences of the more severe forms of IPV. When expressed as relative odds, each five-point difference in the EPDS score was associated with AORs for the outcome ranging from 1.17–1.18 (being slapped or punched) to 1.29 (being attacked with a weapon). While the magnitudes of the coefficients cannot be directly compared, the magnitudes of the test statistics suggest that the strength of the association between IPV and subsequent depression is stronger than the strength of the association between depression and subsequent IPV.

**Table 4:** Association between lagged depression symptom severity and intimate partner violence.

	Index of IPV intensity		Slapped in past year		Shoved in past year		Punched in past year		Attacked with weapon in past year	
	coeff.	95% CI	marg. eff.	95% CI	marg. eff.	95% CI	marg. eff.	95% CI	marg. eff.	95% CI
EPDS score (per 5 points)	0.054	0.030,0.079	0.023	0.015,0.031	0.017	0.009,0.025	0.010	0.004,0.016	0.009	0.004,0.013
Assigned to intervention arm	-0.007	-0.075,0.061	-0.012	-0.042,0.018	-0.004	-0.032,0.023	0.002	-0.019,0.023	-0.003	-0.019,0.012
Age (per 5 y)	-0.011	-0.050,0.028	-0.020	-0.036,-0.005	-0.022	-0.037,-0.007	0.0003	-0.011,0.011	0.004	-0.005,0.012
Household asset wealth										
Poorest	Ref		Ref		Ref		Ref		Ref	
Poorer	0.003	-0.109,0.114	0.008	-0.037,0.052	-0.012	-0.055,0.031	0.013	-0.017,0.043	0.002	-0.022,0.026
Middle	-0.048	-0.162,0.065	-0.004	-0.055,0.047	-0.015	-0.062,0.032	0.026	-0.009,0.061	-0.017	-0.040,0.006
Richer	-0.06	-0.177,0.058	-0.016	-0.066,0.034	-0.031	-0.077,0.016	0.003	-0.032,0.038	-0.011	-0.037,0.015
Richest	-0.06	-0.177,0.056	-0.024	-0.074,0.026	-0.018	-0.067,0.031	-0.001	-0.034,0.033	-0.002	-0.031,0.027
Completed high school	-0.097	-0.206,0.012	-0.045	-0.093,0.002	-0.023	-0.069,0.023	-0.039	-0.080,0.001	-0.008	-0.033,0.016
Time point										
Baseline	Ref		Ref		Ref		Ref		Ref	
6 mo	-0.085	-0.157,-0.012	-0.063	-0.093,-0.034	-0.035	-0.065,-0.006	-0.023	-0.045,-0.001	-0.013	-0.029,0.003
18 mo	-0.07	-0.144,0.004	-0.057	-0.088,-0.026	-0.037	-0.066,-0.007	-0.016	-0.039,0.007	-0.016	-0.033,-0.0001
Employed	-0.048	-0.120,0.024	-0.013	-0.054,0.027	-0.009	-0.045,0.027	-0.026	-0.056,0.003	-0.013	-0.035,0.008
Father of child present	0.041	-0.029,0.112	0.015	-0.015,0.045	0.031	0.001,0.061	0.003	-0.020,0.026	-0.004	-0.021,0.013
HIV serostatus										
HIV-negative	Ref		Ref		Ref		Ref		Ref	
Unknown	-0.038	-0.216,0.141	-0.019	-0.078,0.040	-0.005	-0.063,0.054	-0.017	-0.059,0.025	-0.008	-0.040,0.024
HIV-positive	-0.012	-0.091,0.067	0.009	-0.027,0.044	0.028	-0.005,0.062	0.006	-0.019,0.030	-0.010	-0.027,0.006
AUDIT-C score (per point)	0.06	0.035,0.085	0.021	0.015,0.027	0.015	0.010,0.020	0.010	0.006,0.014	0.006	0.004,0.009
Monthly household income										
0–499 ZAR	Ref		Ref		Ref		Ref		Ref	
500–1,000 ZAR	-0.132	-0.322,0.059	-0.017	-0.085,0.052	-0.019	-0.082,0.045	-0.015	-0.067,0.038	-0.007	-0.036,0.021
1,001–2,000 ZAR	-0.099	-0.288,0.091	-0.026	-0.091,0.039	-0.022	-0.085,0.041	-0.025	-0.075,0.026	0.012	-0.018,0.042
2,001–5,000 ZAR	-0.142	-0.329,0.045	-0.026	-0.091,0.039	-0.029	-0.092,0.034	-0.032	-0.082,0.018	-0.003	-0.031,0.024
5,001–8,000 ZAR	-0.126	-0.337,0.085	-0.027	-0.105,0.050	-0.056	-0.130,0.018	-0.032	-0.095,0.031	-0.011	-0.049,0.026
≥8,000 ZAR	-0.182	-0.391,0.028	-0.066	-0.160,0.029	-0.055	-0.140,0.031	-0.051	-0.117,0.015	0.009	-0.048,0.066
Self-reported diabetes	0.19	-0.144,0.525	0.072	-0.030,0.174	0.029	-0.074,0.132	0.056	-0.014,0.126	0.022	-0.016,0.060
Self-reported hypertension	-0.059	-0.163,0.045	-0.025	-0.078,0.027	-0.015	-0.065,0.035	-0.042	-0.086,0.001	-0.003	-0.029,0.023

AUDIT-C, three-item consumption subset of the Alcohol Use Disorders Identification Test; EPDS, Edinburgh Postnatal Depression Scale; IPV, intimate partner violence; ZAR, South African Rand

## Discussion

In this secondary analysis of data from a population-based, 3-y cluster-randomized controlled trial of more than 1,200 women in peri-urban South Africa, we found that both IPV and depression were highly prevalent and that IPV intensity had a statistically significant association with depression symptom severity. The estimated association was relatively large in magnitude: the experience of IPV was associated with a difference in depression symptom severity that was comparable to the treatment effects observed in short-term randomized-controlled trials of psychotherapy interventions for peripartum depression [69,70]. Our findings are consistent with what has been shown in longitudinal studies conducted among women in high-income countries [23,67,71], plausible in light of what is generally known about the adverse psychological impacts of stressful life events and traumatic stressors [13–17], and robust to alternative specifications and potential confounding by time-invariant factors. Taken together, our findings have important policy and programmatic implications for women’s health in sub-Saharan Africa.

The association between IPV and poor mental health outcomes is generally accepted in the field [20,72], but most of the evidence is based on data from high-income countries. The evidence base from sub-Saharan Africa has lagged. The study in the literature most similar to ours is a study of 1,045 women in northeastern Brazil who were interviewed one time during pregnancy and one time postpartum [73]. In that study, Ludermir and colleagues [73] showed that IPV during pregnancy was associated with increased incidence of postpartum depression. Since the publication of the systematic review by Devries and colleagues [23], in which they identified no longitudinal studies of IPV and incident depression from sub-Saharan Africa, there has been one new study examining the relationship between sexual violence and mental-health–related outcomes among HIV-positive women in rural Uganda [28]. Several factors limit the generalizability of that study. Estimation was limited to HIV-positive women enrolled in a long-term HIV cohort. Given the myriad ways in which HIV stigma undermines treatment uptake [74–76], it is likely that theirs was a fairly unique population that had overcome substantial barriers to remain engaged in care [77,78]. (In contrast, in our sample, less than two-thirds of the HIV-positive women had re-engaged in HIV care following childbirth [68].) Furthermore, the findings of Tsai and colleagues [28] were based on a relatively small sample size and a relatively small number of exposure events. The analysis described in this manuscript is based on data collected from a large, population-based sample of women recruited from the community, thereby overcoming some of the limitations of their work.

Notably, we found that the association between IPV and depression was bi-directional: not only was IPV associated with greater subsequent depression symptom severity but also depression symptom severity was associated with a greater risk of subsequent IPV. Concerns about victim-blaming have hampered empirical research into understanding how individual characteristics of IPV survivors may be predictive of subsequent revictimization [79]. Our findings are consistent with prior conceptual and empirical work describing the role of psychological difficulties in maintaining abusive relationships [26,80,81]. Our findings are also consistent with longitudinal studies conducted with survivors of IPV showing that symptoms of post-traumatic stress are predictive of subsequent revictimization [82–84]. The data do not permit us to understand the mechanisms linking symptoms of depression to increased victimization risk. It is possible, for example, that depression may influence partner selection, reduce self-efficacy for leaving abusive relationships, or lead to distorted cognitions about risk, or some combination of the above. If IPV and depression are intertwined in a vicious cycle, with IPV increasing the risk of future depression and depression increasing the risk of future revictimization, these mutually reinforcing effects could potentially undermine the effectiveness of single-component interventions. It is possible that combined interventions, such as a broad-based package of services (e.g., case management, crisis services, legal aid, transitional housing, and childcare support) [85] plus cognitive-behavioral therapy [27] may be effective in interrupting the cycle of IPV and depression, but the effectiveness of such a multi-component approach is as of yet unknown.

In addition to bringing longitudinal, population-based data to bear on this issue, our analysis makes several unique contributions to this literature. First, the analysis is based on data from a highly vulnerable population: pregnant women living in one of the poorest communities in South Africa, where both the experience of violence and depression are extremely common. Second, our outcome measure has strong evidence of reliability, criterion-related validity, and construct validity in the local setting [39,43–47]. Third, given the richness of the data, our analysis permitted adjustment for potentially confounding factors (that could influence risks for both IPV and depressed mood) noted by Devries and colleagues [23] to have received less attention in the literature, including alcohol abuse [86,87]. Fourth, the fixed effects design accounts for potential confounding by time-invariant factors (that could also influence risks for both IPV and depression), such as personality structure [88] and childhood adversity [60,89,90]. And finally, our quantile regression and fixed effects quantile regression estimates indicate that the adverse impacts of IPV are greatest for women in the upper end of the conditional depression distribution.

Interpretation of our findings is subject to a number of assumptions and limitations. First, the fixed effects regression model relies on within-participant variation over time to estimate the quantities of interest and therefore could not estimate associations between time-invariant factors (e.g., educational attainment) and depression symptom severity. However, this is a small price to pay for the ability to gain greater traction on estimating the primary association of interest, i.e., between IPV and depression. Second, while Hill’s [91] criteria for causation require temporality, the exposure and outcome must be consistent with the known latency period. Most studies in the field have used a latency period of 3–6 mo for observing adverse changes in mental health in response to a stressor [92–94]. In our study, interviews were conducted at baseline, 6 mo, 18 mo, and 36 mo, suggesting an average latency period of 12 mo in the lagged-covariate and fixed-effects specifications. The extended latency period could have caused us to underestimate the strength of the association. However, in many studies in the literature on medical and psychosocial risk factors for depression, extended latency periods are not uncommon and may extend from 12 mo [95] to 2 y or even more [71,96]. Third, exposure assessment in this study was limited to different types of physical violence and, to a lesser extent, emotional violence. The home visiting intervention studied in the parent randomized controlled trial was designed to promote maternal and child health in a broad sense, and violence against women was one of many different aspects of wellness examined [30–35]. To minimize respondent burden, this study did not assess sexual violence or a more comprehensive range of emotional violence. However, these other aspects of IPV tend to co-occur as part of an overall phenotype of controlling behavior [1,67,90,97,98], suggesting a certain degree of collinearity. Therefore, while assessment of these violent behaviors should be incorporated into future studies of IPV (particularly because the adverse impacts of emotional violence on depression may be at least as severe as those resulting from physical and sexual violence [67,73,98,99]), we do not believe that failure to measure these other aspects of IPV would have biased our estimates away from the null. Fourth, outcomes were assessed using a screening instrument rather than structured diagnostic interviews. The EPDS is a reliable, sensitive, and valid instrument for assessing depression symptom severity [44], and its use in studies of partner violence and psychological distress is standard in the field [73]. The limitations of using screening instruments in this context are well known [100]. However, even sub-syndromal symptoms of depression that do not rise to the occasion of a formal diagnosis of major depressive disorder may entail significant psychosocial impairment and should therefore be pertinent to public health interest [101].

These limitations notwithstanding, our findings provide novel, population-based, longitudinal evidence from sub-Saharan Africa corroborating the link between IPV and negative mental health outcomes. The medical and psychological treatment of victimized women can likely be improved by attending to the underlying causes of their symptoms. Universal screening interventions (that seek to identify asymptomatic victimized women) have not proved successful in improving mental-health–or quality-of-life–related outcomes [102–104], but more intensive health sector responses should be explored. For example, screening combined with individually tailored psychosocial intervention has been shown to be effective in reducing health risks among pregnant and postpartum women [105,106]. Because the association between IPV and depression is likely reciprocal, psychosocial interventions may have important collateral impacts on reducing women’s susceptibility to violence [27]. Further research elucidating the conditions under which depression increases women’s susceptibility to IPV may yield additional targets for intervention. Because IPV is frequently embedded within an overall pattern of controlling behavior (“patriarchal terrorism” [107]) that can give rise to these bi-directional relationships, clinicians may need to modify their treatment plans if IPV is part of the complex network of causal influences on their patients’ depression. For women at risk of further victimization, judicious selection of an antidepressant medication without adverse cognitive effects should be considered to minimize the possibility of iatrogenically compromising their ability to respond to or avoid threatening situations or leave abusive relationships [12]. And post-discharge safety plans for women who have attempted suicide will need to involve collaboration with extended social ties (rather than return to family) if partner abuse is suspected as a causative factor. Outside of the health sector, structural interventions [108] that seek to modify the context of gender-unequal norms in which violence against women is overlooked, sustained, or encouraged [20] should also be explored as means of reducing IPV and improving women’s mental health [109–111].

## Supporting Information

S1 STROBE Checklist Checklist of items that should be included in reports of observational studies. (DOC)

S1 Table Association between lagged exposure to intimate partner violence, by type, and depression symptom severity. (DOCX)

S1 Text Analysis history. (DOCX)

## Abbreviations

AOR: adjusted odds ratio
AUDIT-C: three-item consumption subset of the Alcohol Use Disorders Identification Test
CI: confidence interval
EPDS: Edinburgh Postnatal Depression Scale
ICC: intra-class correlation
IPV: intimate partner violence
