# Supplementary material for: Intimate Partner Violence and Depression Symptom Severity among South African Women during Pregnancy and Postpartum: Population-Based Prospective Cohort Study
Source: PLoS Med. 2016 Jan 19;13(1):e1001943. doi: 10.1371/journal.pmed.1001943 (PMC4718639; doi:10.1371/journal.pmed.1001943)
Supplement: S1 STROBE Checklist — (DOC) [file pmed.1001943.s001.doc]

STROBE Statement—checklist of items that should be included in reports of observational studies

|  | Item No | Recommendation |
| --- | --- | --- |
| **Title and abstract** | 1 | (*a*) Indicate the study’s design with a commonly used term in the title or the abstract  **Population-Based Prospective Cohort Study** |
|  |  | (*b*) Provide in the abstract an informative and balanced summary of what was done and what was found  **Done** |
| Introduction | | |
| Background/rationale | 2 | Explain the scientific background and rationale for the investigation being reported  **Intro, Para 1-3: Explained conceptual hypotheses for association between partner violence and depression, with reference to the Foa conceptual model** |
| Objectives | 3 | State specific objectives, including any prespecified hypotheses  **Intro, Para 3** |
| Methods | | |
| Study design | 4 | Present key elements of study design early in the paper  **Done** |
| Setting | 5 | Describe the setting, locations, and relevant dates, including periods of recruitment, exposure, follow-up, and data collection  **Done: Methods, Para 2-3** |
| Participants | 6 | (*a*) *Cohort study*—Give the eligibility criteria, and the sources and methods of selection of participants. Describe methods of follow-up  **Done: Methods, Para 2-3** |
|  |  | (*b*) *Cohort study*—For matched studies, give matching criteria and number of exposed and unexposed  **Not applicable (no matching done)** |
| Variables | 7 | Clearly define all outcomes, exposures, predictors, potential confounders, and effect modifiers. Give diagnostic criteria, if applicable  **Methods, Para 4-7: outcome, explanatory variables, covariate adjusters all explained** |
| Data sources/ measurement | 8* | For each variable of interest, give sources of data and details of methods of assessment (measurement). Describe comparability of assessment methods if there is more than one group  **Done (see above)** |
| Bias | 9 | Describe any efforts to address potential sources of bias  **Methods, Para 9: address bias due to unobserved confounding**  **Methods, Para 10: address bias due to reverse causality** |
| Study size | 10 | Explain how the study size was arrived at  **Sample size was fixed (this was a secondary analysis of data from an RCT)** |
| Quantitative variables | 11 | Explain how quantitative variables were handled in the analyses. If applicable, describe which groupings were chosen and why  **Page 8-9: Outcome and exposure were specified as continuous variables.**  **Page 12: Sensitivity analysis to interrogate the specification of these variables** |
| Statistical methods | 12 | (*a*) Describe all statistical methods, including those used to control for confounding  **Done** |
|  |  | (*b*) Describe any methods used to examine subgroups and interactions  **N/A- there were no subgroup analyses** |
|  |  | (*c*) Explain how missing data were addressed  **Results, Para 1 and Table 1- very little missing data** |
|  |  | (*d*) *Cohort study*—If applicable, explain how loss to follow-up was addressed  **Results, Para 1: LTFU was acceptable, and participants LTFU were roughly comparable** |
|  |  | (*e*) Describe any sensitivity analyses  **Results, Para 6: sensitivity analyses to interrogate specification of outcome and explanatory variables** |

Continued on next page

| Results | | |
| --- | --- | --- |
| Participants | 13* | (a) Report numbers of individuals at each stage of study—eg numbers potentially eligible, examined for eligibility, confirmed eligible, included in the study, completing follow-up, and analysed  **Results, Para 1** |
|  |  | (b) Give reasons for non-participation at each stage  **Readers referred to primary RCT publication** |
|  |  | (c) Consider use of a flow diagram  **Readers referred to primary RCT publication** |
| Descriptive data | 14* | (a) Give characteristics of study participants (eg demographic, clinical, social) and information on exposures and potential confounders  **Results, Para 1 and Table 1** |
|  |  | (b) Indicate number of participants with missing data for each variable of interest  **Table 1** |
|  |  | (c) *Cohort study*—Summarise follow-up time (eg, average and total amount)  **N/A- all participants followed for 36 months** |
| Outcome data | 15* | *Cohort study*—Report numbers of outcome events or summary measures over time  **Results, Para 2** |
|  |  | *Case-control study—*Report numbers in each exposure category, or summary measures of exposure |
|  |  | *Cross-sectional study—*Report numbers of outcome events or summary measures |
| Main results | 16 | (*a*) Give unadjusted estimates and, if applicable, confounder-adjusted estimates and their precision (eg, 95% confidence interval). Make clear which confounders were adjusted for and why they were included  **Results, Para 3** |
|  |  | (*b*) Report category boundaries when continuous variables were categorized  **Results, Para 4: Outcome and exposure were specified as continuous variables but specified as categorical variables in sensitivity analysis** |
|  |  | (*c*) If relevant, consider translating estimates of relative risk into absolute risk for a meaningful time period  **Done- logistic regression estimates transformed into marginal effects** |
| Other analyses | 17 | Report other analyses done—eg analyses of subgroups and interactions, and sensitivity analyses |
| Discussion | | |
| Key results | 18 | Summarise key results with reference to study objectives  **Discussion, Para 1** |
| Limitations | 19 | Discuss limitations of the study, taking into account sources of potential bias or imprecision. Discuss both direction and magnitude of any potential bias  **Discussion, Para 5** |
| Interpretation | 20 | Give a cautious overall interpretation of results considering objectives, limitations, multiplicity of analyses, results from similar studies, and other relevant evidence  **Done** |
| Generalisability | 21 | Discuss the generalisability (external validity) of the study results  **Done** |
| Other information | | |
| Funding | 22 | Give the source of funding and the role of the funders for the present study and, if applicable, for the original study on which the present article is based  **Done** |

*Give information separately for cases and controls in case-control studies and, if applicable, for exposed and unexposed groups in cohort and cross-sectional studies.

**Note:** An Explanation and Elaboration article discusses each checklist item and gives methodological background and published examples of transparent reporting. The STROBE checklist is best used in conjunction with this article (freely available on the Web sites of PLoS Medicine at http://www.plosmedicine.org/, Annals of Internal Medicine at http://www.annals.org/, and Epidemiology at http://www.epidem.com/). Information on the STROBE Initiative is available at www.strobe-statement.org.
