# Supplementary material for: Intimate Partner Violence and Depression Symptom Severity among South African Women during Pregnancy and Postpartum: Population-Based Prospective Cohort Study
Source: PLoS Med. 2016 Jan 19;13(1):e1001943. doi: 10.1371/journal.pmed.1001943 (PMC4718639; doi:10.1371/journal.pmed.1001943)
Supplement: S1 Table — (DOCX) [file pmed.1001943.s002.docx]

Table S1. Association between lagged exposure to intimate partner violence, by type, and depression symptom severity

|  | coeff. | 95% CI | coeff. | 95% CI | coeff. | 95% CI | coeff. | 95% CI | coeff. | 95% CI |
| --- | --- | --- | --- | --- | --- | --- | --- | --- | --- | --- |
| Slapped in past year | 1.84 | 1.05,2.64 |  |  |  |  |  |  | 0.63 | -0.31,1.57 |
| Shoved in past year |  |  | 2.42 | 1.52,3.32 |  |  |  |  | 1.66 | 0.61,2.72 |
| Punched in past year |  |  |  |  | 2.66 | 1.52,3.80 |  |  | 1.29 | -0.14,2.72 |
| Attacked w weapon in past year |  |  |  |  |  |  | 1.14 | -0.37,2.66 | -0.91 | -2.58,0.76 |
| Assigned to intervention arm | -0.11 | -0.74,0.52 | -0.19 | -0.81,0.43 | -0.14 | -0.76,0.49 | -0.15 | -0.79,0.48 | -0.16 | -0.78,0.46 |
| Age (per 5 years) | 0.4 | 0.07,0.72 | 0.42 | 0.09,0.74 | 0.36 | 0.03,0.68 | 0.35 | 0.02,0.68 | 0.42 | 0.09,0.74 |
| Household asset wealth |  |  |  |  |  |  |  |  |  |  |
| Poorest | Ref |  | Ref |  | Ref |  | Ref |  | Ref |  |
| Poorer | -0.78 | -1.78,0.22 | -0.74 | -1.73,0.25 | -0.76 | -1.76,0.23 | -0.78 | -1.79,0.23 | -0.74 | -1.73,0.25 |
| Middle | -1.18 | -2.28,-0.07 | -1.16 | -2.25,-0.07 | -1.17 | -2.26,-0.07 | -1.16 | -2.27,-0.05 | -1.17 | -2.26,-0.08 |
| Richer | -0.97 | -2.05,0.10 | -1.02 | -2.08,0.05 | -1.02 | -2.09,0.05 | -1.03 | -2.11,0.06 | -1 | -2.07,0.06 |
| Richest | -1.3 | -2.36,-0.24 | -1.33 | -2.38,-0.28 | -1.33 | -2.39,-0.27 | -1.39 | -2.45,-0.32 | -1.29 | -2.35,-0.24 |
| Completed high school | -1.51 | -2.54,-0.48 | -1.58 | -2.59,-0.56 | -1.52 | -2.55,-0.49 | -1.6 | -2.64,-0.57 | -1.51 | -2.53,-0.48 |
| Time point |  |  |  |  |  |  |  |  |  |  |
| Baseline | Ref |  | Ref |  | Ref |  | Ref |  | Ref |  |
| 6 months | -0.5 | -1.22,0.22 | -0.61 | -1.33,0.11 | -0.67 | -1.39,0.06 | -0.77 | -1.49,-0.05 | -0.51 | -1.24,0.21 |
| 18 months | -0.41 | -1.15,0.34 | -0.54 | -1.28,0.19 | -0.64 | -1.38,0.11 | -0.79 | -1.53,-0.05 | -0.42 | -1.16,0.33 |
| Employed | -1.11 | -1.82,-0.39 | -1.13 | -1.84,-0.42 | -1.12 | -1.84,-0.40 | -1.16 | -1.88,-0.43 | -1.11 | -1.82,-0.40 |
| Father of child present | -0.63 | -1.33,0.06 | -0.69 | -1.38,-0.00 | -0.61 | -1.30,0.08 | -0.59 | -1.28,0.11 | -0.69 | -1.38,-0.00 |
| HIV serostatus |  |  |  |  |  |  |  |  |  |  |
| HIV-negative | Ref |  | Ref |  | Ref |  | Ref |  | Ref |  |
| Unknown | 1.13 | -0.59,2.85 | 1.01 | -0.70,2.72 | 1.09 | -0.63,2.80 | 1.02 | -0.70,2.74 | 1.08 | -0.63,2.79 |
| HIV-positive | 0.58 | -0.17,1.34 | 0.56 | -0.19,1.31 | 0.59 | -0.16,1.34 | 0.63 | -0.13,1.39 | 0.54 | -0.20,1.29 |
| AUDIT-C score (per point) | 0.08 | -0.09,0.25 | 0.08 | -0.09,0.25 | 0.1 | -0.07,0.27 | 0.14 | -0.03,0.31 | 0.06 | -0.11,0.23 |
| Monthly household income |  |  |  |  |  |  |  |  |  |  |
| 0-499 ZAR | Ref |  | Ref |  | Ref |  | Ref |  | Ref |  |
| 500-1000 ZAR | 0 | -1.68,1.67 | 0.06 | -1.60,1.72 | 0.1 | -1.57,1.76 | 0.03 | -1.64,1.71 | 0.07 | -1.59,1.73 |
| 1001-2000 ZAR | -1.01 | -2.59,0.56 | -0.94 | -2.50,0.61 | -0.93 | -2.51,0.64 | -0.96 | -2.54,0.62 | -0.95 | -2.51,0.61 |
| 2001-5000 ZAR | -1.49 | -3.06,0.09 | -1.47 | -3.02,0.08 | -1.43 | -3.00,0.13 | -1.5 | -3.08,0.07 | -1.43 | -2.99,0.12 |
| 5001-8000 ZAR | -2.37 | -4.11,-0.62 | -2.4 | -4.12,-0.67 | -2.27 | -4.01,-0.53 | -2.32 | -4.07,-0.56 | -2.36 | -4.09,-0.63 |
| ≥8000 ZAR | -2.91 | -4.90,-0.92 | -2.75 | -4.74,-0.77 | -2.87 | -4.85,-0.89 | -2.82 | -4.82,-0.81 | -2.84 | -4.82,-0.86 |
| Self-reported diabetes | 2.68 | -0.48,5.84 | 2.95 | -0.13,6.03 | 2.8 | -0.25,5.86 | 2.77 | -0.41,5.94 | 2.89 | -0.16,5.94 |
| Self-reported hypertension | 1.07 | -0.16,2.30 | 1.07 | -0.16,2.29 | 1.08 | -0.15,2.30 | 1.05 | -0.19,2.28 | 1.09 | -0.13,2.31 |

*AUDIT-C, 3-item consumption subset of the Alcohol Use Disorders Identification Test; EPDS, Edinburgh Postnatal Depression Scale; ZAR, South African Rand*
