# Supplementary material for: Intimate Partner Violence and Depression Symptom Severity among South African Women during Pregnancy and Postpartum: Population-Based Prospective Cohort Study
Source: PLoS Med. 2016 Jan 19;13(1):e1001943. doi: 10.1371/journal.pmed.1001943 (PMC4718639; doi:10.1371/journal.pmed.1001943)
Supplement: S1 Text — (DOCX) [file pmed.1001943.s003.docx]

**S1 Text.** Analysis history for the observational study described in: Tsai AC, Tomlinson M, Comulada WS, Rotheram-Borus MJ. Intimate partner violence and depression symptom severity among South African women during pregnancy and postpartum: population-based prospective cohort study. PLOS Medicine.

We did not publish or pre-register a protocol for this secondary analysis. We followed a clear analysis plan, as described in the methods section. Further details on the analysis history are described below:

1. The study was motivated by a straightforward hypothesis grounded in a previously published review by Devries et al. [1] and our own prior work (e.g., an observational study published by Tsai et al. [2]).

2. The inclusion/exclusion criteria for the study were established at the outset of the study. As described in the manuscript, all women participating in the cluster-randomized controlled trial by Rotheram-Borus et al. [3] were eligible for inclusion in this secondary analysis. No women were excluded. There were therefore no degrees of freedom that could have been manipulated by excluding some study participants.

3. The statistical analyses were determined at the outset and were not changed. However, we did conduct a number of exploratory sensitivity analyses to interrogate the robustness of our findings. As detailed in the manuscript, these were as follows:

(a) Instead of examining exposure to intimate partner violence as a single index, we examined each of the four types of IPV separately. There were therefore no degrees of freedom that could have been manipulated by aggregating the exposure variable.

(b) Instead of examining depression symptom severity as a linear dependent variable, we dichotomized this variable at a known threshold (≥13) based on previous studies in this Xhosa-speaking population [4,5,6,7,8]. The primary analysis relies on the use of the continuous variable. There were therefore no degrees of freedom that could have been manipulated by choosing an arbitrarily cutpoint.

(c) We reversed the ordering of the exposures and outcomes to determine the extent to which the estimated association was bidirectional. This analytic maneuver was identical to that employed in our previous work [2,9].

4. There was some internal discussion among the team members about whether or not to adjust for high blood pressure and diabetes because there was concern that this could represent overadjustment for physical health variables that could lie on the causal pathway between the exposure and outcome. However, it was also argued that these measures of health status could represent legitimate confounders. The decision to *exclude* these variables from the regression models would not have appreciably shifted the primary estimates in any way (e.g., in the primary regression shown in in Table 2, the magnitude of the regression coefficient would have increased from from 1.038 to 1.043, and the t-statistic would have decreased from 4.77 to 4.70). Ultimately we made the decision to retain these variables in the regression models given that they were initially pre-specified in the analysis plan.

5. We did not conduct any subgroup analyses. However, we used quantile regression. Our decision to do so was driven by our *a priori* intention to assess the sensitivity of our finding to shape as well as locational shifts in the distribution, as well as by the more general issue (mentioned by the statistical reviewer) that this technique is under-utilized. No prior studies in the literature on IPV and depression have employed this methodology. Importantly, none of the primary conclusions are driven by the quantile regression results (e.g., we do not focus on specific points in the conditional distribution).

S1 TEXT REFERENCES

1. Devries K, Mak J, Bacchus LJ, Child JC, Falder G, Petzold M, et al. (2013) Intimate partner violence and incident depressive symptoms and suicide attempts: a systematic review of longitudinal studies. PLoS Med 10: e1001439.

2. Tsai AC, Wolfe WR, Kumbakumba E, Kawuma A, Hunt PW, Martin JN, et al. (in press) Prospective study of the mental health consequences of sexual violence among women living with HIV in rural Uganda. J Interpers Violence, in press. Epub 13 Jan 2015. doi:10.1177/0886260514567966

3. Rotheram-Borus MJ, Tomlinson M, Roux IL, Stein JA (2015) Alcohol use, partner violence, and depression: a cluster randomized controlled trial among urban South African mothers over 3 years. Am J Prev Med 49: 715-725.

4. Tsai AC, Tomlinson M, Dewing S, le Roux IM, Harwood JM, Chopra M, et al. (2014) Antenatal depression case-finding by community health workers in South Africa: feasibility of a mobile phone application. Arch Womens Ment Health 17: 423-431.

5. Hung KJ, Tomlinson M, le Roux IM, Dewing S, Chopra M, Tsai AC (2014) Community-based prenatal screening for postpartum depression in a South African township. Int J Gynaecol Obstet 126: 74-77.

6. Rochat TJ, Richter LM, Doll HA, Buthelezi NP, Tomkins A, Stein A (2006) Depression among pregnant rural South African women undergoing HIV testing. JAMA 295: 1376-1378.

7. Honikman S, van Heyningen T, Field S, Baron E, Tomlinson M (2012) Stepped care for maternal mental health: a case study of the Perinatal Mental Health Project in South Africa. PLoS Med 9: e1001222.

8. Rochat TJ, Tomlinson M, Newell ML, Stein A (2013) Detection of antenatal depression in rural HIV-affected populations with short and ultrashort versions of the Edinburgh Postnatal Depression Scale (EPDS). Arch Womens Ment Health 16: 401-410.

9. Tsai AC, Weiser SD, Dilworth SE, Shumway M, Riley ED (2015) Violent victimization, mental health, and service utilization outcomes in a cohort of homeless and unstably housed women living with or at risk of becoming infected with HIV. Am J Epidemiol 181: 817-826.
